# Anaesthetist-controlled versus patient-maintained effect-site targeted propofol sedation during elective primary lower-limb arthroplasty performed under spinal anaesthesia (ACCEPTS): study protocol for a parallel-group randomised comparison trial

**DOI:** 10.1186/s13063-019-3228-4

**Published:** 2019-02-13

**Authors:** David W. Hewson, Frank Worcester, James Sprinks, Murray D. Smith, Heather Buchanan, Philip Breedon, Jonathan G. Hardman, Nigel M. Bedforth

**Affiliations:** 1 Department of Anaesthesia and Critical Care, Nottingham University Hospitals NHS Trust, Nottingham, UK; 2 Medical Design Research Group, Nottingham Trent University, Nottingham, UK; 3 Community and Health Research Unit, University of Lincoln, Lincoln, UK; 4 Division of Rehabilitation and Ageing, University of Nottingham, Nottingham, UK; 5 Anaesthesia and Critical Care, University of Nottingham, Nottingham, UK

**Keywords:** Propofol sedation, Patient-maintained propofol sedation, Regional anaesthesia, Joint arthroplasty, Procedural sedation

## Abstract

**Background:**

The clinical efficacy of effect-site targeted patient-maintained propofol sedation (PMPS) compared to anaesthetist-controlled propofol sedation (ACPS) for patients undergoing awake joint replacement surgery is currently unknown. There is no commercially available medical device capable of delivering PMPS so we have designed and built such a device. We plan a clinical trial to compare PMPS to ACPS and to collect data relating to the safety of our prototype device in delivering sedation.

**Methods:**

The trial is an open-label, randomised, controlled superiority trial recruiting adults who are undergoing elective primary lower-limb arthroplasty with sedation by propofol infusion by effect-site targeting into two equal-sized parallel arms: PMPS and ACPS. The primary research objective is to compare the body-weight-normalised rate of propofol consumption when sedation for surgery on adults undergoing elective primary lower-limb arthroplasty under spinal anaesthesia is patient-maintained versus when it is anaesthetist-controlled. The study primary null hypothesis is that there is no difference in the rate of propofol consumption when sedation is patient-maintained versus anaesthetist-controlled.

**Discussion:**

This is the first trial to test the superiority of effect-site-targeted patient-maintained propofol sedation versus anaesthetist-controlled propofol sedation in terms of total propofol consumption during the sedation period. The results of this trial will help inform clinicians and device manufacturers of the clinical efficacy and safety of patient-maintained propofol sedation applied to a common operative setting.

**Trial registration:**

International Standard Randomised Controlled Trial Number Registry, ISRCTN29129799. Prospectively registered on 12 June 2018.

**Electronic supplementary material:**

The online version of this article (10.1186/s13063-019-3228-4) contains supplementary material, which is available to authorized users.

## Background

The most commonly performed lower limb surgical arthroplasty procedures in the UK are primary hip replacement and primary knee replacement; respectively, 93,234 and 102,519 replacements were performed in 2016 (excluding Scotland) [1]. Regional anaesthesia, consisting of a spinal anaesthetic with or without peripheral nerve blockade, provides excellent operating conditions and post-operative analgesia, reduces the risk of venous thromboembolism, reduces transfusion requirements in the peri-operative period and avoids some of the risks associated with general anaesthesia [2].

Some patients are willing to experience surgery awake, but a significant proportion of patients are fearful of this experience pre-operatively and experience varying degrees of anxiety before and during the operation [3, 4]. Anaesthetists commonly supplement spinal anaesthesia with sedation medication delivered intravenously for lower limb arthroplasty in anxious patients. The aim of sedation is to reduce anxiety and promote psychological comfort, while minimising the physiological side effects induced by deeper planes of sedation.

Sedation for lower limb arthroplasty is often provided by the supervising anaesthetist using propofol by intravenous computer-assisted target-controlled infusion (anaesthetist-controlled propofol sedation (ACPS)) [5]. A target-controlled infusion (TCI) device delivers a variable rate of propofol in order to obtain and maintain a specified effect-site (i.e. brain) drug concentration, until such time as a new concentration is set by the supervising anaesthetist. Their use is standard anaesthesia practice in the UK.

There is, however, an unpredictable individual dose response to drugs inducing sedation, even using modern ACPS with TCI systems. Furthermore, anaesthetists have been shown to be poor judges of the pre-operative anxiety states of individual patients [6]. For a variety of reasons, including anxiety itself, patients do not always have preconceived expectations about how sedated they wish to be for surgery and these expectations are not always clearly communicated to (or understood by) the treating anaesthetist.

For these reasons it is difficult for anaesthetists to sedate each individual to their preferred level using ACPS. Under-sedation results in an anxious patient because their anxiolytic requirements are unmet during surgery. Over-sedation results in a patient receiving an unnecessarily deep level of sedation and heightened exposure to the associated physiological harms even though the patient would have willingly experienced surgery at a lighter plane of sedation.

Patient-maintained propofol sedation (PMPS) involves the use of a TCI system delivering propofol where the effect-site concentration is influenced by the patient using a handheld trigger. Allowing patients to influence the depth of their sedation with a propofol TCI system has already been described in small case series in healthy volunteers [7] and in general [8], dental [9] and ambulatory outpatient surgery [10]. There are only three randomised controlled trials of PMPS and none of these use the Schnider effect-site-targeted propofol TCI model [5] to compare PMPS against ACPS [11–13]. Our aim is to assess the clinical efficacy of PMPS compared to ACPS. There is no commercially available device to perform PMPS, previous studies having relied on prototype devices. We have therefore designed and built a device capable of delivering PMPS for use in this clinical trial.

## Methods/design

A standard protocol items: recommendation for interventional trials (SPIRIT) checklist detailing the items addressed in this clinical trial protocol is provided as Additional file 1.

### Study objectives

The primary research objective is to compare the body-weight-normalised rate of propofol consumption when sedation for surgery on adults undergoing elective primary lower-limb arthroplasty under spinal anaesthesia (effect-site-targeted with propofol) is patient-maintained versus when it is anaesthetist-controlled. The primary outcome will be expressed as milligrams propofol per kilogram actual body weight per hour of the sedation period. The sedation period is defined as the time from commencement of the allocated sedation regimen by the supervising clinical anaesthetist (which will be after spinal blockade is confirmed by clinical dermatomal testing) until the sedation regimen is discontinued at the end of surgery (which will be at the application by the operating surgeon of clips to skin).

The study primary null hypothesis is that there is no difference in the rate of propofol consumption when sedation it is patient-maintained versus anaesthetist-controlled. The primary alternative hypothesis is that a patient-maintained propofol sedation regimen will result in a minimum 29% reduction in the rate at which propofol is consumed over the course of the sedation period compared to anaesthetist-controlled propofol sedation.

The secondary research objectives are:

- To establish whether patients are less deeply sedated intra-operatively during patient-maintained sedation compared to anaesthetist-controlled sedation.
- To assess whether patients undergoing PMPS have equal reductions in peri-operative anxiety compared to patients undergoing ACPS.
- To observe whether patients undergoing PMPS have comparable post-operative satisfaction with their sedation experience compared to patients undergoing ACPS.
- To explore whether patients who have undergone PMPS are fit for discharge from the post-anaesthetic care unit (PACU) sooner than patients who have undergone ACPS.
- To determine if patients undergoing PMPS have calculated effect-site concentrations of propofol that are lower than in patients who undergo ACPS.
- To examine how many times patients using PMPS press their trigger button to increment their dose (successful button press), how many times they press the button while the device is locked out (unsuccessful button press) and whether there is a relationship between pre-operative anxiety state and use of the trigger system.
- To determine whether patients undergoing PMPS experience a different incidence of airway, respiratory or cardiovascular sedation-related side-effects than patients who have undergone ACPS. Airway sedation-related side-effects include partial or complete airway obstruction that requires the usual clinical anaesthetist to apply one of the following interventions: chin-lift, jaw thrust, nasopharyngeal airway insertion, oropharyngeal airway insertion, laryngeal mask insertion or endotracheal tube insertion. Respiratory sedation-related side-effects are respiratory rate fewer than 8 breaths per minute or arterial oxygen saturations less than 88% in patients with chronic obstructive pulmonary disease (COPD) or 94% in all other patients. Cardiovascular sedation-related side-effects include heart rate or blood pressure reduction greater than 20% from baseline, but such events may be related to spinal anaesthesia rather than the sedation. All such events will be noted by a study investigator and reviewed to determine the likely aetiology. There are no objective clinical criteria to determine this, but routine clinical practice is to assimilate the presented information to estimate the likely contribution from sedation, spinal anaesthesia and/or another cause.
- To determine whether patients undergoing PMPS experience different health-related quality of life outcomes compared to patients undergoing ACPS.

### Study design and setting

The trial is an open-label, randomised, controlled superiority trial recruiting adults who are undergoing elective primary lower-limb arthroplasty with sedation by propofol infusion by effect-site targeting into two equal-sized parallel arms: PMPS and ACPS. The study will be run as a single-centre study at Nottingham University Hospitals National Health Service (NHS) Trust. The trial will be conducted only in a secondary care setting.

### Trial intervention

Propofol 1% is licensed in the UK for “Sedation for diagnostic and surgical procedures, alone or in combination with local or regional anaesthesia in adults and children > 1 month” [14]. The trial will be using propofol within the terms of this license. Patients randomised to PMPS will receive propofol according to the following algorithm: the effect-site target concentration of propofol will be commenced at 0.5 μg.mL^− 1^ and increased by 0.2 μg.mL^− 1^ (when the patient presses the button) to a maximum of 2.0 μg.mL^− 1^. Following a successful button-induced increase in the effect-site target, further button presses will not increase the target concentration for 2 min (this is termed the *lockout period*). If patients do not press the button for 15 min, the effect-site target will reduce by 0.1 μg.mL^− 1^, and will continue to reduce by 0.1 μg.mL^− 1^ every 15 min to a minimum of 0.5 μg.mL^− 1^ in the absence of a button-press. Patients randomised to ACPS will receive propofol according to the following algorithm: the effect-site concentration will be commenced at a level determined by the supervising clinical anaesthetist and incremented and decremented by them as they see fit. No maximum or baseline levels will be pre-specified. This constitutes standard clinical practice. Both groups will use Schnider effect-site TCI modelling.

### Safety

All pre-existing medication taken by a participant as part of their usual medical care will be permitted. Co-administration of sedatives, hypnotics or analgesics during placement of spinal anaesthesia or during the intra-operative sedation regime at the discretion of the supervising clinical anaesthetist will be recorded. Behavioural disinhibition is a well-recognised side-effect of propofol sedation. This occurs regardless of whether the propofol is delivered by intermittent bolus, fixed rate infusion or target-controlled infusion. It manifests as patient movement (usually of the upper limbs) and/or talking. It does not always respond readily to simple verbal command from the supervising clinician to desist. The standard treatment for this is for the supervising clinical anaesthetist to actively alter the depth of sedation at their discretion by either lightening or deepening the degree of sedation according to clinical circumstances and their professional judgement. In either arm of the trial, if a patient displays disinhibited behaviour that is disruptive to surgery or potentially dangerous for the patient (for example, risking pulling out their intravenous cannula), the supervising clinical anaesthetist will be free to alter the patient’s depth of sedation either by lightening or deepening sedation, using a sedative agent of their discretion. This will be recorded. No medications will be specifically prohibited in this trial.

In order to detect physiological changes that may suggest unsafe sedation, both trial arms will have heart rate, respiratory rate, arterial oxygen saturations, blood pressure and depth of sedation measured and recorded at 5 min intervals throughout the sedation period. Adverse events, adverse device events, serious adverse events and serious adverse device events will be recorded and reported to the relevant bodies (trial Sponsor, device manufacturer, Research Ethics Committee, Medicine and Healthcare products Regulatory Agency).

### Inclusion criteria

The inclusion criteria are as follows:

- Listed to undergo elective primary hip or knee arthroplasty under spinal anaesthesia
- Expressing a pre-operative preference for sedation during surgery
- Able to communicate in written and spoken English
- Capable of giving informed consent
- Age > 18 years

### Exclusion criteria

- Allergy to propofol
- Male patients with body mass index (BMI) > 42 and female patients with BMI > 37
- Medical contraindication to spinal anaesthesia (for example local infection at injection site, patient refusal, allergy to local anaesthetic agent, untreated systemic infection or untreated coagulopathy)
- Expressing pre-operative preference for surgery to be performed awake or under general anaesthesia
- Inability to use handheld trigger system of the patient-maintained propofol sedation device (PMPSD)
- Pregnancy or breastfeeding

BMI is included as an exclusion criterion since in the Schnider TCI model, lean body mass (LBM) is calculated using the James formula. This has been shown to estimate LBM satisfactorily in normal-weight and moderately obese patients, but paradoxically in male patients with BMI > 42 and female patients with BMI > 37 [15]. As a safety precaution to prevent inaccurate propofol dosing in these groups we have excluded patients with BMI above these thresholds.

### Enrolment

Patient screening for eligibility and recruitment will be conducted at the Theatre admission lounge, Nottingham City Hospital campus. This site is where our patients are usually treated. Screening will be performed by the supervising clinical anaesthetist during their routine pre-operative patient assessment. The supervising clinical anaesthetist will notify a study investigator of potentially eligible patients. The recruited patient will sign and date the approved version of the informed consent form before any procedures specific to the clinical investigation are performed.

The participant’s research data will be gathered during a single visit to hospital, which forms part of usual clinical care. A telephone consultation between a study investigator and each participant will be performed at post-operative day 7–10.

After enrolment, the participant will be given a group-specific laminated educational leaflet explaining their sedation system. Those allocated to PMPS will receive written instructions on the use of the PMPSD and those allocated to ACPS will receive written explanation on how their supervising clinical anaesthetist will sedate them.

A pre-operative questionnaire will be administered to all participants before they are taken to the anaesthetic room. The following data will be recorded: age, gender, whether hip or knee arthroplasty, surgeon initials, supervising clinical anaesthetist initials, patient weight and height, American Society of Anaesthetist Physical Status Classification, respiratory rate, arterial oxygen saturations, heart rate, blood pressure (systolic, diastolic, mean) and sedation score on the Modified Observer’s Assessment of Alertness/Sedation (mOAA/S) Scale.

### Intervention

The usual clinical care of non-invasive physiological monitoring will be established by the supervising clinical anaesthetist. They will insert an intravenous cannula and spinal anaesthesia will be performed. When satisfied that adequate spinal anaesthesia has been accomplished, they will commence the participant on their allocated sedation regime.

Participants allocated to PMPS will have their sedation infusion commenced at the minimum level of 0.5 μg.mL^− 1^ and will be given the handheld trigger button to press. They will be told *“*Press the button if you want to be more sleepy*”*. Participants allocated to ACPS will be commenced on an effect-site-steered TCI propofol infusion. The supervising clinical anaesthetist will control the effect-site target at their discretion.

Both sedation regimes will commence in the anaesthetic room, prior to the participant moving to theatre. A study investigator will record physiological and sedation outcome measures at 5-min intervals throughout the sedation period.

The sedation regime will be discontinued at the end of surgery when skin clips are applied to the wound. This time will be noted by a study investigator. In the PMPS group the handheld button will be withdrawn from the patient and the TCI infusion stopped. In the ACPS group the TCI infusion will be stopped. If, for any reason, the sedation is discontinued prior to end of surgery, the reason will be noted in a free-text narrative response by a study investigator.

### Post-operative period

Participants will be transferred to the PACU where a study investigator will continue physiological and sedation outcome measures until readiness for discharge from PACU is achieved. The modified Aldrete score will be recorded at 5-min intervals from the time the sedation regime stops until the time at which the participant is scored in excess of 9, indicating readiness for safe discharge from PACU. Once participants are ready for discharge from PACU a study investigator will administer a short, group-specific post-operative questionnaire including questions on psychological variables (e.g. anxiety).

On post-operative day 7–10 (the exact timing depending on the availability and convenience of the participant), a study investigator will contact all patients by telephone to conduct a group-specific post-operative telephone questionnaire (including questions on anxiety since the operation and the sedation experience) and a brief structured interview (in order for us to gain more in-depth data on the experience and perceptions of the sedation experience for patients in the PMPS group). The interview will include qualitative responses in order to allow the participant to provide additional information beyond that covered in the questionnaires.

**Fig. 1:**
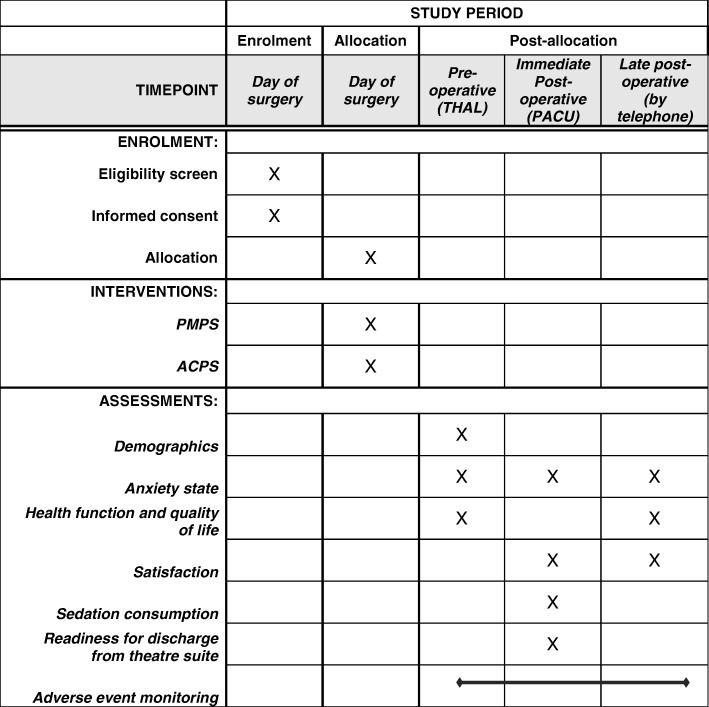
SPIRIT: Schedule of enrolment, intervention and assessments. THAL, Theatre admission lounge; PACU, Postoperative care unit; PMPS, patient-maintained propofol sedation; ACPS, anaesthetist-controlled propofol sedation)

On completion of the post-operative telephone questionnaire the participant’s enrolment in the trial will cease. A SPIRIT schedule of trial enrolment, intervention and assessment is shown in Fig. 1.

### Statistical methods

Propofol consumption (milligrams) will be calculated in participants randomised to the two study groups. Body weight (kilograms) and height (centimetres) recorded at the pre-operative assessment will be used. Length of sedation is defined as the time from when the propofol sedation is commenced by the supervising clinical anaesthetist up until the time when the first skin clip is inserted to close the wound (at which point the anaesthetist will stop the sedation).

The alternative hypothesis is two-sided. The level of statistical significance is 5%. The clinical superiority of PMPS over ACPS will be shown if the mean rate is reduced by 29% (or more) when using PMPS, compared with ACPS. The randomisation schedule will be computer-generated using an 8-block design, with allocation advised by opaque sealed envelope. The expected recruitment rate into the study is 2 patients per week.

### Sample size calculation

Our calculation is based on results from a pilot PMPS study (*n* = 26) and prospectively gathered standard care ACPS (*n* = 17), showing that the mean body-weight-normalised rate of propofol consumption in the PMPS and ACPS groups was 1.580 (SD = 0.755) and 2.231 (SD = 0.915), respectively. For power of 90% and level of significance of 5% against the two-sided alternative hypothesis, 72 participants are required to detect the observed difference in mean rate using Welch’s two-sample *t* test. Expecting 10% drop-out of participants, a sample size of 80 is required. Participants who drop out from the trial prior to randomisation will be removed from the study and any trial data that may have been collected on them or from them will be destroyed. All participants dropping out after randomisation will be included in the study by intention to treat and all trial data collected up to the moment of drop out will be retained for analysis.

### Randomisation

Enrolled participants will be randomised by block randomisation technique. An opaque sealed envelope randomisation system will be used. Allocation concealment will be achieved by the generation of an unpredictable allocation sequence into sealed envelopes by an independent party, which will not be opened until patient consent for inclusion has been obtained. This will be accessed by a study investigator for the purposes of allocation. Randomisation will be conducted on the day of surgery.

### Statistical plan

All data collected will be summarised for reporting purposes using descriptive statistics. The hypothesis test associated with the primary outcome will compare the two mean consumption rates using standard statistical tests chosen appropriate to underlying assumptions (e.g. two-sample *t* test and the Wilcoxon-Mann-Whitney test). Testing will be performed using datasets formed according to inclusions based on intention to treat. *P* values will be reported, with values less than 5% declared statistically significant.

We will report analyses of secondary outcomes for complete cases that contain no major clinical investigation plan violations. Amongst these, button-press data will be modelled using count data regression methods, with extension to bivariate models to distinguish between successful presses and unsuccessful presses. The panel time-series of propofol consumption rates collected throughout the sedation period alongside button presses will together be modelled using marked-point process methods. Ordinal responses recorded on a Likert scale (peri-operative anxiety, patient satisfaction) will be compared across trial arms using parametric methods suitable to underlying assumptions and non-parametric methods, such as the Wilcoxon rank sum test. Preoperative and post-operative quality of recovery total scores will be compared using the Wilcoxon matched pairs test. Inter-item correlations will be computed and associations between scored items will be compared using Spearman rank correlation. The Bonferroni correction to allow for multiple comparisons will be applied. Qualitative data obtained from structured questions will be coded and computer-analysed. Any missing outcome data will be imputed using either a last-observation-carried-forward rule or, for the subset of comparable secondary outcomes, use data routinely measured and recorded as part of usual clinical care.

Health economic analysis aims to establish the net monetary benefit due to the NHS from introducing PMPS compared to the current practice of ACPS. Economic modelling will rely on parameterisations developed from the data gathered from all trial outcome measures, including health-related quality of life from the Euroqol (EQ)-5D-5 L questionnaire before/after data. These data will be collected as part of the pre-operative and post-operative participant interviews. Cost differentials across the two trial arms are expected due to propofol use and time to fitness for discharge from PACU.

No interim analyses are planned for this study. Any deviation from the original statistical plan will be described and justified in the final report.

## Discussion

### Trial blinding

Trial participants cannot usefully be blinded to their intervention, since one group (PMPS) will be asked to use an additional medical device (a trigger button) whereas the other group (ACPS) will not. Some previous trials have attempted to achieve participant blinding in trials of patient-maintained sedation, by giving the control group (ACPS) a sham button to press. This blinding technique has the potential to affect outcome measures in the ACPS group thus we will not be using this blinding technique. Some of the potential psychological benefit of patient-maintained sedation is that patients are able to exert control over their care. By giving patients in the control arm (ACPS) a button to press, this could alter their psychological response to surgery and sedation and provide additional anxiolysis and comfort, or possibly the reverse. Additionally, the action of giving a sham button to patients in the ACPS group, means they are no longer receiving what would be considered normal anaesthetist-controlled propofol sedation.

The participant’s usual clinical team (anaesthetist, surgeon, operating department staff) will not be blinded to the intervention and will be independent from the trial team. The supervising clinical anaesthetist will, for the reasons outlined above, be impossible to blind, since they will be delivering sedation directly to participants allocated to the ACPS group. A blinding technique giving both group participants a trigger button (a sham one in the ACPS group) and both group participants an ACPS infusion device (a sham one in the PMPS group) would be very difficult logistically in a theatre environment, be unlikely to provide effective blinding, would alter the psychological responses to surgery and sedation in both groups reducing the validity of comparison between groups and provide little benefit when it comes to assessing and reducing bias in the outcome measures we are proposing.

The study investigator who is allocated to collect intra-operative data will not be blinded to the allocation groups. Recording of physiological data will be taken from the usual intra-operative monitoring system in place in theatre, which is in close physical proximity to the participant and their infusion devices. It would not be realistic to blind the observer to this monitoring.

### Trial status

This protocol is version number 2.0 dated 2 August 2018. Recruitment is planned to commence on 18 September 2018 and be completed by 1 February 2020. The trial Sponsor is Nottingham University Hospitals NHS Trust (researchsponsor@nuh.nhs.uk).

## Additional file

Additional file 1: SPIRIT 2013 checklist: recommended items to address in a clinical trial protocol and related documents*. (DOC 121 kb)

## Abbreviations

ACPS: Anaesthetist-controlled propofol sedation
BMI: Body mass index
mOAA/S: Modified observer’s assessment of alertness/sedation scale
NHS: National Health Service
PACU: Post-anaesthesia care unit
PMPS: Patient-maintained propofol sedation
PMPSD: Patient-maintained propofol sedation device
TCI: Target-controlled infusion
UK: United Kingdom
